# Parainfluenza virus infection associated with posterior reversible encephalopathy syndrome: a case report

**DOI:** 10.1186/1752-1947-6-89

**Published:** 2012-03-26

**Authors:** Owolabi Ogunneye, Jaime A Hernandez-Montfort, Yetunde Ogunneye, Iheanyichukwu Ogu, Daniel Landry

**Affiliations:** 1 Department of Medicine, Baystate Medical Center, Tufts University School Of Medicine, 759 Chestnut Street, Springfield, MA 01199, USA

**Keywords:** posterior reversible encephalopathy syndrome, parainfluenza virus, hypertension

## Abstract

**Introduction:**

Posterior reversible encephalopathy syndrome is a clinical and radiological entity. The most accepted theory of posterior reversible encephalopathy syndrome is a loss of autoregulation in cerebral blood flow with a subsequent increase in vascular permeability and leakage of blood plasma and erythrocytes, producing vasogenic edema. In infection-associated posterior reversible encephalopathy syndrome, a clinical pattern consistent with systemic inflammatory response syndrome develops. Parainfluenza virus has not been reported in the medical literature to be associated with posterior reversible encephalopathy syndrome.

**Case presentation:**

We report herein the case of a 54-year-old Caucasian woman with posterior reversible encephalopathy syndrome associated with parainfluenza virus infection who presented with generalized headache, blurring of vision, new-onset seizure and flu-like symptoms.

**Conclusion:**

Infection-associated posterior reversible encephalopathy syndrome as well as hypertension-associated posterior reversible encephalopathy syndrome favor the contribution of endothelial dysfunction to the pathophysiology of this clinicoradiological syndrome. In view of the reversible nature of this clinical entity, it is important that all physicians are well aware of posterior reversible encephalopathy syndrome in patients presenting with headache and seizure activity. A detailed clinical assessment leading to the recognition of precipitant factors in posterior reversible encephalopathy syndrome is paramount.

## Introduction

Posterior reversible encephalopathy syndrome (PRES) is a clinical and radiological entity first described by Hinchey *et al. *in 1996 [1] and later by Baizabal-Carvallo *et al. *[2]. The common clinical features are headache, seizures, visual changes, altered mental status and focal neurological signs [3]. In a case series of 13 patients described by Varaprasad *et al.*, headache and seizures were the most common manifestations [4].

Imaging is essential in the differential diagnosis of PRES, which includes hypertensive encephalopathy, uremic encephalopathy, lupus cerebritis, meningitis, encephalitis, stroke and cerebral venous thrombosis. Magnetic resonance imaging (MRI) allows more precise characterization and recognition of PRES than computed tomography does [5]. The most common characteristic imaging pattern in PRES seen on MRI is the presence of edema involving the white matter of the posterior portions of both cerebral hemispheres. This is especially true in the parieto-occipital regions, where it appears in a relatively symmetrical pattern that spares the calcarine and paramedian parts of the occipital lobes [6]. However, the brainstem, cerebellum, frontotemporal cortex and basal ganglia may also be involved. Cortical involvement was observed in 95.5% of patients in one case series [2].

In 20% to 30% of patients who develop PRES, blood pressure is essentially normal at the time of presentation, a finding that has been reported in many large series, including those concerning women with eclampsia, as well as in patients with cyclosporine toxicity after allogeneic bone marrow transplant, in broader PRES studies and in many isolated case reports [7].

In adult patients, more than 90% have antibodies to parainfluenza virus. Even in the presence of high levels of serotype-specific antibodies, primary infection and reinfection can occur [8]. Parainfluenza virus is associated with 1% to 15% of acute respiratory illness in adults [9].

## Case presentation

A 54-year-old Caucasian woman presented to the emergency department (ED) of our institution with uncontrolled hypertension and new-onset seizures. Two weeks prior to this admission the patient's hypertensive medication had been switched in a tapered fashion from clonidine to lisinopril because of a reported intolerance in the form of general malaise, sinus headache and non-productive cough. Five days before presentation she had developed generalized headache, blurred vision and nausea, and she decided to visit her closest ED, an outside facility in Northwestern Massachusetts, which found her hypertensive with systolic blood pressure of 260 mmHg. At that time, she was treated with analgesics and parenteral antihypertensive medications, which led to partial improvement of her symptoms and subsequent discharge to her home in rural western Massachusetts. Twenty-four hours before her hospital admission, she again visited the ED of the same outside facility complaining of recurrent, severe headaches accompanied by blurred vision. Her blood pressure recorded at that time was 260/160 mmHg. She was again treated with intravenous narcotics and antihypertensive's; however, while being treated, she developed confusion, and the decision was made to transfer the patient to our hospital. En route to our hospital she had an episode of tonic-clonic seizure activity lasting three to four minutes. In our ED, she received a dose of 1000 mg intravenous fosphenytoin once upon arrival and was subsequently intubated for airway protection because of unresponsiveness. Her admission blood pressure was 194/115 mmHg, and she was given two doses of 10 mg of intravenous labetalol.

Her medical history was notable for hypertension, systemic lupus erythematosus (SLE), end-stage renal disease (ESRD) secondary to lupus nephritis on daily ambulatory intermittent peritoneal dialysis, with a history of good dialysis compliance and blood pressure control. Her most recent peritoneal dialysis treatment had been the day preceding admission. She had also had a mechanical aortic valve replacement for a history of aortic stenosis and underwent chronic anticoagulation treatment. She was no longer on any immunosuppressive therapy for lupus. She did not use alcohol, tobacco or recreational drugs. There were no reported sick contacts, recent travel or outdoor exposures.

**Figure 1:**
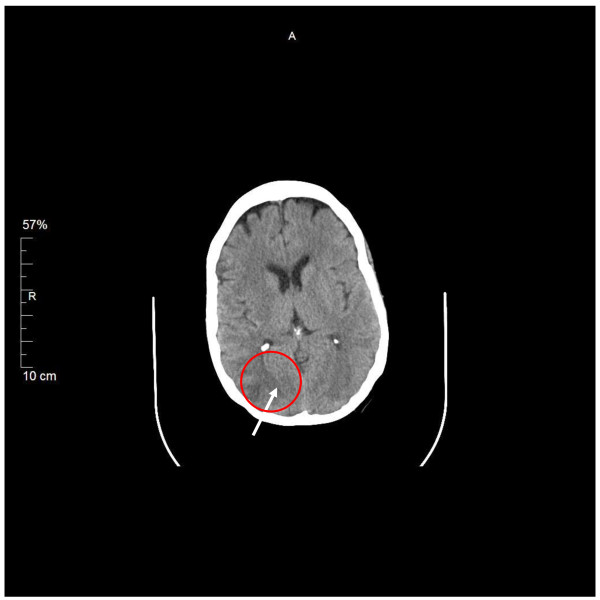
**Computed tomographic image showing findings in the brain of a patient with posterior reversible encephalopathy syndrome**. Subtle evidence of a possible acute abnormality in the right occipital lobe (arrow) in addition to an unusual pattern of calcification in the cerebral hemispheres.

Her initial laboratory data were relevant for leukocytosis of 16,500 cells/mm^3^. No thrombocytopenia or schistocytes were noted on peripheral blood smear. Her liver panel was normal. Her blood urea nitrogen was 68 mg/dl with serum creatinine of 5.6 mg/dl. Her baseline serum creatinine was 5.2 mg/dl. Continuous cycling peritoneal dialysis was initiated in the Intensive-Care Unit (ICU). She underwent computed tomography of the brain, which showed subtle evidence of a possible acute abnormality in the right occipital lobe in addition to an unusual pattern of calcifications in the cerebral hemispheres (Figure 1). The patient underwent lumbar puncture for cerebrospinal fluid (CSF) analysis, which showed clear fluid, a white blood cell count of 99 cells/mm^3^, a protein level of 9860 mg/dl and a glucose level of 53 mg/dl. Her chest X-ray was unremarkable. Empiric treatment for viral and bacterial meningitis with intravenous acyclovir, vancomycin and ceftriaxone followed. Her blood and CSF cultures ultimately showed no growth, and her CSF venereal disease research laboratory, herpes simplex and varicella virus polymerase chain reaction results were negative.

In our ICU, the physical examination revealed an afebrile patient with a blood pressure of 145/92 mmHg while sedated on propofol and intubated on assist control ventilation, tidal volume of 550 ml, positive end-expiratory pressure of 5 cmH_2_O, oxygen saturation of 100%, fraction of inspired oxygen of 50% and heart rate of 85 beats/minute. She had a noticeable generalized fine maculopapular rash not reported on admission. Her head and neck examination revealed no signs of trauma, with equal and reactive pupils without subconjunctival hemorrhage. Her neck was supple without lymphadenopathy. Her neurological examination showed no obvious posturing or asymmetry; however, she had bilateral lower-extremity hyperreflexia accompanied by the presence of a coexistent Babinski sign. Her lungs were clear to auscultation bilaterally, and her cardiac examination showed a regular pulse with normal S1 and S2 and an audible systolic click. No rashes on her palms or soles were noted. No embolic or immunologic phenomena of endocarditis were encountered.

**Figure 2:**
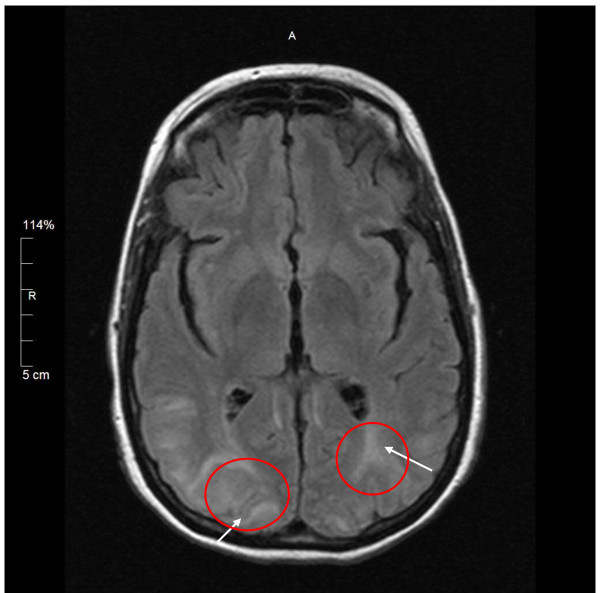
**MRI findings in the brain of a patient with posterior reversible encephalopathy syndrome**. Multifocal, T2-weighted, hyperintense lesions are present within the parasagittal posterior parietal lobe, both cerebellar hemispheres, and the occipital lobes bilaterally (arrows).

On ICU day 2, the patient developed a fever of 102°F, and a nasopharyngeal direct fluorescent antibody test was positive for parainfluenza virus, which was confirmed by viral cultures for parainfluenza virus type 3. Lupus activity studies showed normal C3/C4 levels and elevated complement 50 levels > 60 U/ml. Her anti-nuclear antibody titer was 1:800 with a homogeneous nucleolar pattern. Transesophageal echocardiography showed normal prosthetic valve function and no evidence of vegetations. MRI of the brain without contrast enhancement was also performed on ICU day two. The image revealed multifocal T2-weighted hyperintense lesions present within the parasagittal white matter and both cerebellar hemispheres (Figure 2).

## Discussion

The most accepted theory of PRES is a loss of autoregulation in cerebral blood flow induced by hypertension with a subsequent increase in vascular permeability and leakage of blood plasma and erythrocytes, producing vasogenic edema with petechial hemorrhage [1,2]. A histologic evaluation of PRES is uncommon and is often obtained late in the course of complex systemic disease. Activated reactive astrocytes, scattered macrophages and lymphocytes have often been noted without inflammation, ischemia or neuronal damage. Three hemispheric pattern variants may be encountered with similar frequency: holohemispheric, superior frontal sulcal and primary parietal-occipital [10].

PRES has been less commonly described in the setting of autoimmune disease, and the distinctive role of SLE in the pathophysiology of PRES is often clouded by concurrent hypertension and renal disease [11]. To date, there have been two types of "PRES-SLE" syndromes described: "hypertensive PRES," which is reversible with conventional antihypertensive and anticonvulsive treatments concomitant with inactive SLE, and "immunological PRES," which requires immunosuppressive therapy and is considered a neurological manifestation of active SLE [12]. Our patient likely had the former type of PRES-SLE, given the fact that markers of lupus activity were normal and her clinical findings were not consistent with cerebral vasculitis or a systemic disease process.

In infection-associated PRES, a clinical pattern consistent with systemic inflammatory response syndrome develops with evidence of multiple organ dysfunction syndrome [10]. A case of an association between influenza A virus and PRES with documentation of cerebral vasculopathy has been reported [13]. Influenza infection can precipitate encephalopathy, or encephalitis with the development of cerebral edema, which might confuse the initial clinical picture. The case of our patient is unique in that parainfluenza virus infection has not been reported in the literature in association with the development of PRES. Parainfluenza infections are a common cause of respiratory infection in infants and children; however, immunocompromised adults can also be affected [14]. Parainfluenza virus type 3 usually presents with lower respiratory features (bronchiolitis and pneumonia), which might have been related to this patient's history of flu-like symptoms that triggered the discontinuation of clonidine, likely representing the incubation period of the virus, which can extend up to seven days. No rash has been reported to be associated with parainfluenza virus [14]. In a study of hematopoietic cell transplant recipients by Peck *et al.*, asymptomatic parainfluenza virus infection was detected in 17.9% of recipients [15].

PRES in our patient was likely precipitated by multiple factors, including uncontrolled hypertension, immunosuppression due to a history of autoimmune disease and ESRD, and acute parainfluenza virus infection.

In addition to viral infection and SLE, PRES has also been associated with high-dose methylprednisolone therapy, uremic encephalopathy, pheochromocytoma, Henoch-Schönlein purpura, acute hepatic failure, sickle cell disease, thrombotic thrombocytopenic purpura, organ transplantation, HIV infection and gemcitabine therapy [3,6].

There are no specific guidelines for the management of hypertension in PRES; however, the general recommendation is to treat all PRES patients presenting with malignant hypertension with parenteral agents for a goal reduction in diastolic blood pressure to 100 to 105 mmHg within two to six hours while not exceeding a decline in mean arterial pressure by more than 25% to avoid cerebral hypoperfusion and stroke. Those patients who present with lesser degrees of hypertension should be managed with smaller incremental changes in blood pressure decline as per ischemic stroke guidelines [16]. Treatment of PRES generally includes antihypertensive and/or anticonvulsant therapies and the withdrawal of a suspected drug if applicable [17]. When seven-day therapy for hypertension and convulsion does not reverse the manifestation, immunosuppressive treatments are recommended to reverse PRES [12]. Recurrence of atypical PRES in a hypertensive child with ESRD on peritoneal dialysis was reported by Girişgen *et al.*, and they noted infection and sudden increases in blood pressure as causes for recurrence [18]. In the case series reported by Varaprasad *et al.*, the duration of hospital stay ranged from five to 26 days (median of 14 days) and the time to recovery from PRES was two to 10 days (median of three and a half days) [4]. Most case reports have described excellent outcomes; however, one report by Covarrubias *et al. *reported mortality of more than 25% with significant residual neurologic morbidity in survivors as well [19].

## Conclusion

Our patient was treated symptomatically, with particular attention given to aggressive blood pressure control. She recovered fully with no residual neurological deficit and with resolution of the generalized body rash, which was ultimately attributed to an allergic reaction. She was discharged to home nine days after admission to our institution.

We postulate that our patient experienced an episode of coexistent hypertensive SLE and infection-associated PRES precipitated by a combination of uncontrolled hypertension and parainfluenza virus type 3 infection. Both associations favor the contribution of endothelial dysfunction in the pathophysiology of this clinicoradiological syndrome.

In view of the reversible nature of this clinical entity, it is important that all physicians are well aware of PRES in patients who present with headache and seizure activity. A detailed clinical assessment leading to the recognition of precipitant factors in PRES is paramount, as early diagnosis and management may have significant prognostic implications.

## Consent

Written informed consent was obtained from the patient for publication of this manuscript and any accompanying images. A copy of the written consent is available for review by the Editor-in-Chief of this journal.

## Competing interests

The authors declare that they have no competing interests.

## Authors' contributions

OO was a major contributor to analyzing and interpreting the data in this case, reviewing the literature and writing the manuscript. JH was a major contributor to the coordination, design and revision of the manuscript critically for important intellectual content. YO was a major contributor to reviewing the literature and developing the images. IO was a major contributor to reviewing the literature. DL was a major contributor to critically reviewing and revising the manuscript for important intellectual content. All authors read and approved the final manuscript.

## References

[B1] Hinchey J Chaves C Appignani B Breen J Pao L Wang A Pessin MS Lamy C Mas JL Caplan LR Reversible posterior leukoencephalopathy syndrome N Engl J Med 1996 334 494 500 10.1056/NEJM199602223340803 8559202

[B2] Baizabal-Carvallo JF Barragán-Campos HM Padilla-Aranda HJ Alonso-Juarez M Estañol B Cantú-Brito C García-Ramos G Posterior reversible encephalopathy syndrome as a complication of lupus activity Clin Neurol Neurosurg 2009 111 359 363 10.1016/j.clineuro.2008.11.017 19128872

[B3] Gopalakrishnan CV Vikas V Nair S Posterior reversible encephalopathy syndrome in a case of postoperative spinal extradural haematoma: case report and review of literature Asian Spine J 2011 5 64 67 10.4184/asj.2011.5.1.64 21386948 PMC3047900

[B4] Varaprasad IR Agrawal S Prabu VN Rajasekhar L Kanikannan MA Narsimulu G Posterior reversible encephalopathy syndrome in systemic lupus erythematosus J Rheumatol 2011 38 1607 1611 10.3899/jrheum.101308 21572160

[B5] Staykov D Schwab S Posterior reversible encephalopathy syndrome J Intensive Care Med 2012 27 11 24 10.1177/0885066610393634 21257628

[B6] Marrone LC Marrone BF de la Puerta Raya J Gadonski G da Costa JC Gemcitabine monotherapy associated with posterior reversible encephalopathy syndrome Case Rep Oncol 2011 4 82 87 10.1159/000324581 21475595 PMC3072184

[B7] Bartynski WS Posterior reversible encephalopathy syndrome, part 2: controversies surrounding pathophysiology of vasogenic edema AJNR Am J Neuroradiol 29 1043 1049 10.3174/ajnr.A0929 PMC8118813 18403560

[B8] Piedra PA Englund JA Glezen WP Richman DD, Whitley RJ, Hayden FG Respiratory syncytial virus and parainfluenza viruses Clinical Virology 2002 2 Washington, DC: ASM Press 763 790

[B9] Falsey AR Walsh EE Viral pneumonia in older adults Clin Infect Dis 2006 42 518 524 10.1086/499955 16421796 PMC7107847

[B10] Bartynski WS Posterior reversible encephalopathy syndrome part 1: fundamental imaging and clinical features AJNR Am J Neuroradiol 29 1036 1042 10.3174/ajnr.A0928 PMC8118828 18356474

[B11] Fugate JE Claassen DO Cloft HJ Kallmes DF Kozak OS Rabinstein AA Posterior reversible encephalopathy syndrome: associated clinical and radiologic findings Mayo Clin Proc 2010 85 427 432 10.4065/mcp.2009.0590 20435835 PMC2861971

[B12] Fujieda Y Kataoka H Odani T Otomo K Kato M Fukaya S Oku K Horita T Yasuda S Atsumi T Koike T Clinical features of reversible posterior leukoencephalopathy syndrome in patients with systemic lupus erythematosus Mod Rheumatol 2011 21 276 281 10.1007/s10165-010-0386-3 21225443

[B13] Bartynski WS Upadhyaya AR Boardman JF Posterior reversible encephalopathy syndrome and cerebral vasculopathy associated with influenza A infection: report of a case and review of the literature J Comput Assist Tomogr 2009 33 917 922 10.1097/RCT.0b013e3181993a43 19940660

[B14] Pickering LK Baker CJ Kimberlin DW Long SS Parainfluenza viral infections Red Book: 2009 Report of the Committee on Infectious Diseases 2009 28 Elk Grove Village, IL: American Academy of Pediatrics 485 487

[B15] Peck AJ Englund JA Kuypers J Guthrie KA Corey L Morrow R Hackman RC Cent A Boeckh M Respiratory virus infection among hematopoietic cell transplant recipients: evidence for asymptomatic parainfluenza virus Blood 2007 110 1681 1688 10.1182/blood-2006-12-060343 17502457 PMC1975849

[B16] Vaughan CJ Delanty N Hypertensive emergencies Lancet 2000 356 411 417 10.1016/S0140-6736(00)02539-3 10972386

[B17] Leroux G Sellam J Costedoat-Chalumeau N Le Thi Huong D Combes A Tieulié N Haroche J Amoura Z Nieszkowska A Chastre J Dormont D Piette JC Posterior reversible encephalopathy syndrome during systemic lupus erythematosus: four new cases and review of the literature Lupus 2008 17 139 147 10.1177/0961203307085405 18250139

[B18] Girişgen I Tosun A Sönmez F Ozsunar Y Recurrent and atypical posterior reversible encephalopathy syndrome in a child with peritoneal dialysis Turk J Pediatr 2010 52 416 419 21043390

[B19] Covarrubias DJ Luetmer PH Campeau NG Posterior reversible encephalopathy syndrome: Prognostic utility of quantitative diffusion-weighted MR images AJNR Am J Neuroradiol 2002 23 1038 1048 12063238 PMC7976914

